# Classical Hodgkin’s Lymphoma in the Era of Immune Checkpoint Inhibition

**DOI:** 10.3390/jcm8101596

**Published:** 2019-10-02

**Authors:** Valli De Re, Laura Caggiari, Ombretta Repetto, Lara Mussolin, Maurizio Mascarin

**Affiliations:** 1 Department of Research and Advanced Tumour Diagnostics, Immunopathology and Tumour Biomarkers Unit/Bio-proteomics Facility, Centro di Riferimento Oncologico di Aviano (CRO), IRCCS, National Cancer Institute, 33081 Aviano, Italy; lcaggiari@cro.it (L.C.); orepetto@cro.it (O.R.); 2 Department of Women’s and Children’s Health, Clinic of Paediatric Haemato-Oncology, University of Padua, 35127 Padua, Italy; lara.mussolin@unipd.it; 3 Institute of Paediatric Research—Fondazione Città della Speranza, 35127 Padua, Italy; 4 Pediatric Radiotherapy and AYA Unit, Centro di Riferimento Oncologico di Aviano (CRO), IRCCS, 33081 Aviano, Italy; mascarin@cro.it

**Keywords:** Hodgkin lymphoma, PD-1, PD-L1, EBV, cytokines, extracellular vesicles, adverse events, angiogenesis, immune checkpoint inhibitors

## Abstract

The ligation of programmed cell death 1 (PD-1) with programmed cell death ligand PD-L activates the immune checkpoint leading to T-cell dysfunction, exhaustion, and tolerance, especially in Hodgkin lymphoma (HL) where the PD-L/ Janus kinase (Jak) signaling was frequently found altered. Anti-PD-1 or anti-PD-L1 monoclonal antibodies can reverse this immune checkpoint, releasing the brake on T-cell responses. The characterization of the mechanisms regulating both the expression of PD-1 and PD-L and their function(s) in HL is ongoing. We provide in this review the recent findings focused on this aim with special attention on the major research topics, such as adverse events and resistance to PD-1–PD-L1 inhibitor treatment, together with a part about angiogenesis, extracellular vesicles, and microbiome in HL pathogenesis.

## 1. Hodgkin’s Lymphoma

Hodgkin’s lymphoma (HL) is a largely curable malignancy of the lymphatic system. Patients usually present with painless lymphadenopathy, although a variety of systemic and organ-specific symptoms may also exist. The diagnosis is based on the identification of the classical Reed–Sternberg cells (HRS) or HRS variants (i.e., in nodular lymphocyte predominant, NLPHL, subtype) in a definite clinical and pathological context. 

There are two types of HL: the NLPHL and the classic HL (cHL). NLPHL is a rare type of HL having a well-recognized morphologic feature of a nodular and/or diffuse proliferation of scattered lymphocyte predominant (LP) tumor cells, set against a background of reactive lymphocytes reminiscent of a primary follicle [1]. NLPHL has typical clinical characteristics and, differently from cHL, has good overall prognosis. cHL is subclassified into four pathologic subtypes (i.e., nodular sclerosis, NSHL; mixed-cellularity, MCHL; lymphocyte-rich, LRHL; and lymphocyte-depletion LDHL) based upon HRS morphology and the cellular composition of the reactive infiltrate in lymph node biopsy specimens [2]. In the differential diagnosis between NLPHL and cHL, immunohistochemistry based on B- and T-cell markers (CD15 and CD30) is of limited help, since there are not specific HL markers.

**Figure 1:**
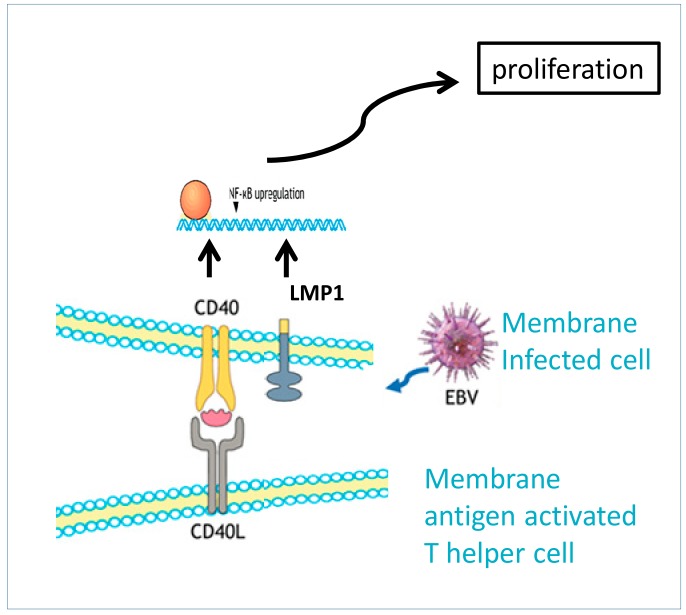
B-cells and Reed–Sternberg cells (HRS) express on their own membrane the CD40 receptor, which binds the CD40 ligand (CD40L) on activated helper T cells. The interaction activates a signal cascade leading to B-cell proliferation through nuclear kappa-light-chain-enhancer of activated B cells (NF-kB)-mediated effector functions. CD40/CD40L interaction also activated deaminase enzyme, responsible for the Ig class exchanges. The Epstein-Barr virus (EBV) latent membrane protein 1 (LMP1) is expressed in EBV latency II stage, which is a characteristic of Hodgkin lymphoma (HL). LMP1 acts as a dysregulated mimic of CD40, inducing enhanced cell activation and survival.

HRS cells are the hallmark of cHL. They are pluri-nucleated giant cells with prominent nucleoli. The origin of these cells has been a topic of several debates for a long time, as they lose the expression of lineage-specific markers (CD20-negative, CD30-positive, and CD15 sometimes). A recent sequencing study classified HRS cells as being derived from pro-apoptotic centrocyte B-cells of the germinal center redirecting to centroblasts, but, differently from their physiological counterpart, this event is not transitory [3]. In a subset of cases, mainly of MCHL subtype, Epstein–Barr virus (EBV) infection can contribute to the distinct cHL molecular signature of lineage-inappropriate gene expression. In these cases, EBV shows a distinctive intermediate type II latency characterized by the expression of latent membrane protein 1 (LMP1). LMP1 protein acts as a mimic of the costimulatory CD40 protein, which activates and sustains different molecular pathways including B cell survival (Figure 1). Notably, EBV is found in HRS cells in nearly all cases of cHL in immunosuppressed patients with HIV infection [4,5]. The particularly high number of immune cells present in the cHL tumor microenvironment play an active role in sustaining the disease [6]. Analysis of the immune cells has identified CD4-positive T-cells as the predominant cell population, which is polarized towards a PD-1-positive Th1 phenotype rather than Th2 effector T-cells and PD-1-negative regulatory T-cells, thus leading to an immunosuppressive microenvironment. PD-L1 expression predominate on tumor-associated macrophages (TAMs), which physically surround PD-L1-positive HRS cells in an immune privileged microenvironmental niche [7]. 

## 2. PD-1/PD-L1 Immune Checkpoint in cHL

Normally, cytotoxic T-cells recognize and kill infected or abnormal tumor cells. To prevent T-cells from damaging healthy and essential cells, several immune system checkpoints exist, where undesirable immune responses can be inhibited or blocked. One inhibitory immune checkpoint (IC) is the programmed cell death 1 (PD-1) pathway. A tumor can activate this IC to protect itself from attacks by the immune system. The first generation of antibody-based inhibitory IC enables to restore a correct immune response towards tumor cells, includes antibodies against cytotoxic T-lymphocyte antigen 4 (CTLA4), programmed cell death (PD-1), and programmed cell death ligands (PD-L1/PD-L2). These IC inhibitors usually have less a minor toxicity than chemotherapy. Their efficacy in the management against non-small-cell lung carcinoma and melanoma had changed the treatment strategies, resulting now in a standard of care for these pathologies. Inhibitory ICs are also effective against other tumors, encompassing HL. In the last years, encouraged by this early success, the indications for IC inhibitors, used alone or in combination, has continued to expand [8]. 

**Figure 2:**
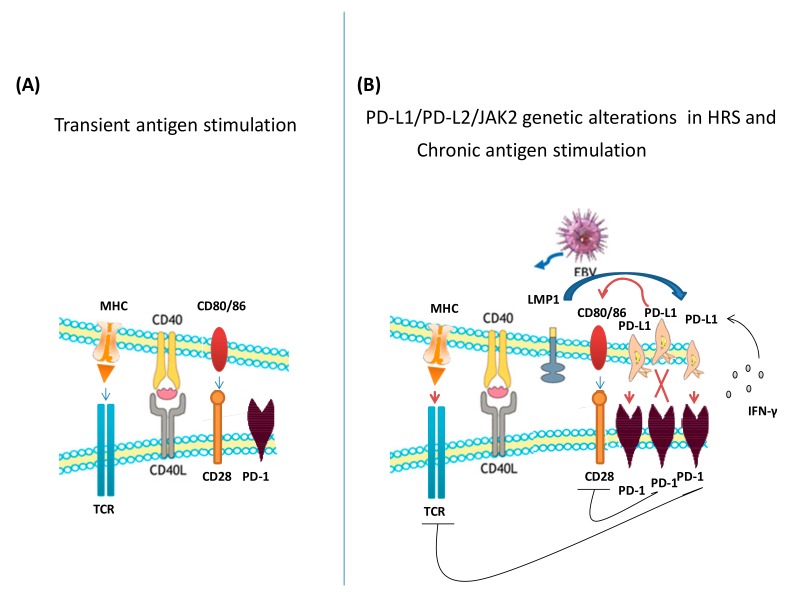
Programmed cell death 1 (PD-1) expression on T-cells (**A**) PD-1 expression is induced when the T-cell receptor (TCR) recognizes an antigen during differentiation from a naïve cell into an effector cell. In the absence of TCR signaling, PD-1 is lost, (**B**) PD-1 expression is maintained upon chronic antigen stimulation such as during chronic viral/bacterial infections and in cancer. When PD-1 binds the programmed cell death 1 ligand (PD-L1) it inhibits TCR/ Major Histocompatibility Complex (MHC) and CD80/CD28 signaling and therby inhibits T cell activation. PD-1–PD-L1 binding occurs predominantly in Hodgkin lymphoma (HL) due to frequent genetic alterations leading to up-regulation of PD-L1/PD-L2/ Janus kinase 2 (JAK 2) signaling or to expression of the Epstein-Barr virus (EBV) latent membrane protein 1 (LMP1) protein that induces the expression of PD-L1 in the Hodgkin Reed-Sternberg (HRS) cells. PD-L1 molecules can also bind the receptor CD80, thus attenuating the signaling from PD-1–PD-L1 interaction.

PD-1 is the transmembrane programmed cell death 1 protein (also called PDCD1 or CD279), mainly produced by activated T-, B-, and myeloid cells. PD-1 expression on naïve T-cells is induced upon T-cell receptor (TCR) activation. In the absence of TCR signaling, its expression is lost while it is maintained upon chronic antigen stimulation such as during chronic viral or bacterial infection and in most cancers (Figure 2) [9]. PD-1 has two ligands, namely PD-L1 (PD-1 ligand 1, CD274, or B7-H1) and PD-L2 (PD-1 ligand 2, CD273, or B7-DC), which are present on the surface of several cell types. The interaction of PD-1 with PD-L1 or PD-L2 inhibits TCR activation, thus preventing immune T-cell proliferation, cytokine production, and adhesion [10,11]. Many tumor cells upregulate PD-L on their surface, thus inhibiting T-cells that might otherwise attack them. Recently, in addition to a link with PD-1, PD-L1 was found to interact with the CD80 co-stimulator T-cell receptor, resulting in the disruption of the PD-L1/PD-1 binding on mature primary dendritic cells, in the restriction of PD-1-positive T-cell function, and in a exacerbation of autoimmunity [12]. These findings revealed the potential of CD80 to increase T-cell activation not only trough CD28 costimulation, but also by reducing the PD-1-driven coinhibitory signal (Figure 2). Both PD-L1 and PD-L2 expression can be induced by exposure to interferons (IFNs), in particular, IFN-γ [13]. However, while PD-L1 is mainly regulated by the IFN-γ signaling converged on the binding of nuclear interferon regulatory factor 1 (IRF-1) to the PD-L1 promoter, PD-L2 is regulated by both IFN-β and IFN-γ, trough both STAT3 and IRF-1 binding to its promoter. Moreover, PD-L2 is expressed on inflammatory macrophages by induction of the alternative Th2 cytokine IL-4 while PD-L1 expression resulted after exposure of lipopolysaccharide (LPS) that generate mainly a Th1 response [14]. PD-1 has a higher binding affinity for PD-L2 (dissociation constant, Kd=140 nM) than for PD-L1 (Kd = 770 nM) [15]. In addition PD-L1, but not PD-L2, shows an affinity also for the costimulatory molecule CD80 with an intermediate affinity between that showed for CTLA-4 and CD28. PD-L1 and PD-L2 are both widely expressed on leukocytes and nonlymphoid cells mostly after pro-inflammatory cytokines exposure and hypoxia, however PD-L2 was more expressed on monocytes and dendritic antigen presenting cells [16]. Overall the difference between PD-L1 and PD-L2 should be reflected in different biological effects and functions in regulating Th1 and Th2 responses. For example, in mice PD-L1, but not PD-L2 associated with the development of autoimmune diabetes [17]. The comparison of PD-L1 and PD-L2 functions is still under investigation and, to date, few studies have examined the correlations of different types of immune cell infiltrates with the prognostic and therapeutic significance of PD-L2 in human cancers [18,19,20,21,22]. A detailed knowledge of PD-L regulation should help identify patients who will not respond to PD-1 blockade therapy. 

The HRS malignant cell in cHL shows a high expression of PD-L1/PD-L2 due to a particularly high frequency of PDL1/PDL2/JAK2 genetic alterations [23] and a correlation with EBV-positive status, mediated by LMP1 (Figure 2) [24].

## 3. PD-1/PD-L1 Inhibitors for cHL Treatment 

Therapeutic antibodies binding to either PD-1 or PD-L1 and therefore, blocking PD-1/PD-L1 interaction may allow the T-cells to attack the tumor (reviewed in [25]). The first drug Nivolumab, in 2014, received approval from the US Food and Drug Administration (FDA) and from the European Medicines Agency (EMA) for treatment of therapy-resistant cHL [26]. Moreover, although they are currently excluded from all trials, recent studies suggest that also patients suffering from autoimmune diseases or who reject transplantation may be eligible for PD-1/PD-L1 immune checkpoint inhibition [27], for example, for CTLA-4 blockade (e.g., in rheumatoid arthritis or in renal transplant patients). Subsequently, other drugs targeting the PD-1/PD-L1 interaction have been developed (Table 1).

The highest antitumor activities of single-agent PD-1 blockade therapy have been observed in carcinogen-induced cancers or malignancies driven by viral infections or showing microsatellite-instability high (MSI-H), which expose a high number of antigens and/or neo-antigens, such as see in EBV-positive) cHL. Anti-PD-1 monotherapy showed the greatest efficacy in relapsed or refractory cHL, which frequently has a high number of PD-1-positive tumor-infiltrating T cells [28]. The response rates can reach 50% to 80% in these situations [29].

Specifically, to cHL, in May 2016 nivolumab received the first approval for the “treatment of patients with cHL that has relapsed or progressed after autologous hematopoietic stem-cell transplantation and post-transplantation brentuximab vedotin” (CheckMate-205 and CheckMate-039) [30].

In March 2017, pembrolizumab was approved for the treatment of adult and pediatric patients with refractory cHL, or patients who relapsed after three or more prior lines of therapy, based on the phase II KEYNOTE-087 trial [31]. In cHL, pembrolizumab showed a lower frequency of adverse events than nivolumab (was 6.4% to 16% vs 22% to 41%of grade 3 to 4, respectively).

However, only a fraction of cHL patients responds to the treatment with anti-PD-1 antibodies (65–87%) [32]. At present, many efforts are; thus, focused on determining the molecular mechanisms at the basis of the resistance to checkpoint blockade. In line with this approach, current efforts are underway to improve clinical responses by incorporating PD-1 inhibitors into earlier treatment regimens and to identify drug classes that better synergize with PD-1 inhibitors, and achieve a lower adverse events or a complete response rates. Thus far, combinations that include anti-PD-1 antibodies are not more effective than anti-PD-1 antibodies alone [33]. However since patients classified as low-risk (iPET-negative) and treated with chemotherapy alone or with chemotherapy and radiation showed a 3 years risk of progression/relapse of ~1% to 10% [34], these patients could benefit from IC inhibitors as they could excludes or at least retard disease relapse. In the future, advanced technologies and better knowledge of the PD-1/PD-L1 signaling mechanism and tumor biology may help oncology closer to the idealized goal of tumor cure without radiotherapy. However, this goal is still far away, while more accessible it is the potential benefit of the use of PD-1-PD-L1 inhibitors and radiotherapy in combination that might change treatment decisions in the coming years. Indeed, several studies demonstrated the ability of radiotherapy to prime and modulate tumor immune response with clinical benefit. One of the most convincing pieces of evidence of the synergy between radiation and immune checkpoint inhibition was obtained in the KEYNOTE-001 trial of pembrolizumab for small-cell lung cancer [35]. In this study the subset of patients treated with pembrolizumab (anti-PD-1) and had radiation at some point in their oncological treatment, had a better survival (PFS and OS) than patients treated with pembrolizumab alone. Indeed, it is well known that radiation could induce an inflammatory response, which can cause tumor necrosis of and promote tumor-associated antigen presentation that activates T-cell immunity. Radiotherapy also enhances immune cell infiltration of tumor by upregulating the expression of adhesion molecules on endothelial cells and the secretion of cytokines that can recruit cytotoxic T lymphocytes. By contrast, high-dose radiation may have an immunosuppressive effect by (i) directly killing radiosensitive CD8-positive effector T lymphocytes, (ii) inducing the production of transforming growth factor-beta (TGF-β) and colony-stimulating factor-1 (CSF-1), and (iii) increasing the expression of PD-L1 and PD-1 in tumor cells and T lymphocytes, respectively. There are evidences that the balance between the two opposing immune reactions may be dependent on the radiation regimen (dose and treatment volume), and the alternative schedule of radiotherapy and immune checkpoint inhibitors. Therefore, of fundamental importance is the design and the conduction of randomized clinical trials of radiotherapy with and without PD-1–PD-L1 inhibitors, which will help to establish whether radiotherapy is clinically beneficial in the setting of PD-1–PD-L1 therapy and vice versa. In recent years, promising results have been reported using radio-chemotherapies in addition to IC inhibitors in treating refractory HL (e.g., [36,37]).

## 4. Resistance to PD-1 and PD-L1 Inhibitors and the Search for Predictive Biomarkers

A correlation has been observed between the expression of PD-L1 in tumor tissue and the likelihood of the response to blockade therapy in various malignancies where pre-existing immunity is presumably suppressed by PD-L1. However, it is getting evident that the PD-1 and PD-L1 expression levels, tested by immunohistochemistry, although approved by FDA, are insufficient to decide to use PD-1/PD-L1 inhibitor treatment decision. For instance, some patients, although presenting high PD-L1 expression, did not respond to the PD-L1 inhibitors, while other patients whose tumor were PD-L1 negative had objective responses [38]. Indeed, it is now clear that in HL, 9p24.1 genetic alterations and high PD-1 or PD-L1 expression, show significant but not absolute correlation with the overall treatment response and that other factor(s) are necessary for the determination of drug efficacy. Must attention has been paid to PD-L1 expressed not only on tumor cells but also on tumor-infiltrating immune cells (TILs), particularly myeloid antigen-presenting cells (e.g., macrophages and myeloid dendritic cells) [39,40]. Indeed, despite the presence of a high number of immune cells, some tumors may be immune unresponsive. The exact mechanism for this resistance is so far not completely understood, although it is presumable that different epigenetic and genetic factors present in the tumor cells and/or in the tumor microenvironment cooperate in this phenomenon. Indeed, mutations negatively affecting the antigen processing, antigen presentation and recognition, or immune cell migration, may lead to a malfunctioned immunity [41,42]. For example, the loss of beta-2 microglobulin (B2M) expression reduces the cell surface expression of the major histocompatibility complex (MHC) class I protein, thereby impairing antigen presentation to cytotoxic T-cells [43]. The loss of phosphatase and tensin homolog (PTEN) expression up-regulates immunosuppressive chemokines like CCL2, which inhibits macrophage polarization from M1 to M2 and VEGF expression. Thus by inducing changes in effector immune cells and blood vessel permeability and architecture, these alterations contribute to recruit immunosuppressive cells and blocking tumor-reactive T-cells [44]; alterations in Wnt/β-catenin signaling causes a decrease in the production of the chemoattractant chemokine CCL4, which leads to a decreased infiltration of natural killer and dendritic cells in the tumor microenvironment [45]. Moreover, epigenetic modifications can modify the expression of genes involved in several important pathways, such as those involved in (i) chromatin modifiers (e.g., the polycomb repressive complex 2, PRC2, and the histone demethylating, H3K27me3) and (ii) Th1-type chemokine production (i.e., those leading to CXCL9, CXCL10, and IFN signatures) [46,47,48]).

In regard to more specifically-malignant HRS cells, results were particularly significant: (i) A decrease in several MHC molecules such as b2M/MHC-class I (79% to 83.2%), MHC-class II (46.8% to 67%) [49], and HLA-G [50,51]; (ii) PD-L1/PD-L2/Jak genetic alterations; (iii) EBV LMP1 expression; and (iv) high PD-1 expression in infiltrating T-cells. B-cells, the precursor of HRS cells, usually express MHC class II and costimulatory CD80/CD86 molecules that are functionally active, allowing these cells to act as antigen-presenting cells by themselves, thus a difference in the expression of these markers has been associated with poor prognosis in CHL patients treated with conventional therapies. As an emerging finding, both MHC-associated antigen presentation and CD80/PD-L1 interaction are involved in the resistance to both conventional and immune therapies through the modulation of PD-1/PD-L1 T-cell activation in B-cells [12,28]. Therefore, a defective function of the PD-1/PD-L1 interaction in antigen presentation and/or effector T-cell functions arise as relevant for the immune checkpoint inhibitory response in cHL, including the involvement of the secreted forms of PD-1 and PD-L1 [7,52]. 

These studies underline the important role of inflammation in the HL tumor microenvironment in the response(s) to therapy, which includes the CD28 expression on TILs, the CD80/CD86 level, the PD-L2 expression, and the stage of JAK/STAT signaling in HRS, among others. Improving understanding of the molecular mechanisms involved in the pathogenesis of HL may help to the development of more accurate predictive algorithms and identification of novel predictive marker(s). Many molecular and clinical studies now underway aim to this. 

Based on results obtained, the updated strategies currently available to select patients who would benefit from immune treatment are the same for all tumors treated with IC inhibitors and include some tests for the determination of: (i) serum levels of IFN-γ, a cytokine that can potentially induce PD-L1 expression and indirectly reflects the effector T-cell activity [53]; (ii) the tumor mutational burden (TBM) defined as the total number of nonsynonymous mutations (e.g., point mutations, frame-shift, insertion or deletion) per coding area of a tumor genome [54]; and (iii) the number of CD8-positive and the density of monocytes TILs on the tumor sample [55]. To improve the predictive value of serum IFN-γ test, it is possible to combine the IFN-γ concentration analysis with the IFN-stimulated chemokine (i.e., CXCL9, CLCL10, and CXCL11) or with analysis of the effector cytokines reflecting a lower immune response (e.g., IL-10, IL-35, IL-4, TGF-β). For instance, data can be made more accurate by adding to the test the number of cell types associated with immunesuppressive activity, e.g., the number of myeloid-derived suppressor cells, inactivated M2 macrophages, or regulatory T cells (Treg).

In December 2017, the FDA approved use of the next-generation FoundationOne test to assess the states of TMB in patients affected by solid (FoundationOne CDX) or hematological and sarcoma tumors (FoundationOne Heme) as a predictive marker of response to anti-PD-1/PD-L1 therapy. The test plans the whole sequencing of 315 genes for solid tumors and 406 genes for either hematological tumors or sarcoma. Although many efforts are now performed to reduce the number of selected genes necessary for the response to ≤100 genes, so far the test remains costly and time-consuming to be applied as a standard clinical test in most clinical centers. The focus of several research topics is represented by TMB-estimating algorithms in a cancer-specific manner and the decrease in cost and time required for the TMB assessment.

## 5. Adverse Effects

The use of IC inhibitors had led to imbalance in immune responses resulting in several adverse events remembering several aspects of autoimmune and inflammatory diseases [56,57]. Usually, these adverse events are transitory but, in some cases, they can be severe and live-threatening [58,59,60,61]. The incidence and severity (e.g. grade, duration) of these adverse events vary among the different classes of IC inhibitors [62,63,64,65], and in accordance with their use in drug combinations [66]. 

Adverse events of grade 2 lead to the interruption of the treatment and to the necessity of a systemic supportive treatment (e.g., corticosteroids). Grade 3 and 4 are differentially treated depending on the organ involved (e.g., skin (pruritus, rash, maculopapular rash, vitiligo, and dermatitis), digestive tract (colitis, diarrhea, pancreatitis, and increased AST/ALT/bilirubin), lung, endocrine glands (hypothyroidism, hyperthyroidism, hypophysitis, thyroiditis, and adrenal insufficiency), nervous system (pneumonitis, lung infiltration, and interstitial lung disease), heart (myocarditis), and urinary system (increased creatinine, nephritis, and renal failure)). 

According to a meta-analysis [67], in patients with various malignancies treated with PD-1 inhibitors, the overall incidence of adverse events of any grade was 26.82%, while for severe grade it was 6.10%; the rate of toxicity-induced death was 0.17%. However, the toxicity of these drugs increases by up to 50% when they are used in combination with other drugs. A review [68] summarized the different aspects and the proper management of each one of these specific toxicities. Adverse events reported during nivolumab treatment in cHL include: fatigue, infusion-related reactions, and rash in about 15% of patients; neutropenia and increased lipase (grade 3–4) in 5%; and serious pyrexia in 4% [30]. 

Of note, as experience with PD-1 and PD-L1 inhibitors grows, there are anecdotal reports of a particular phenomenon of rapid disease progression called “hyperprogression”, which suggests that in a subset of patients IC blockade has the opposing effect of accelerating the disease [69]. This effect is not specific for anti-PD-1 and anti-PD-L1 agents, but they have already been described for other therapeutic immune agents. It is supposed that, depending on tumor cell genetic alterations, this treatment might activate alternative signaling networks leading to the enhancement of tumor growth [70]. Moreover, some compensatory mechanisms could be induced by immune pathways, besides to PD-1/PD-L1, which could in turn favor some tumor escape mechanisms (e.g. local inflammation, angiogenesis, matrix and tissue remodeling, metabolism modification) [70].

## 6. Angiogenesis and Extracellular Vesicles

A link between cancer and thrombosis was established several years ago [71,72,73]; thrombosis is the second-leading cause of death in hematologic malignancies [74]. Coagulation homeostasis is often altered in patients with cancer [73], including patients with HL [75]. Proteomic analysis of plasma from patients with relapsed HL showed an up-regulation of important proteins involved in the coagulation process, like fibrinogen and complement C3; a reduction of coagulation inhibitors like antithrombin and α-1-antitrypsin (also named serpin), and HRS possess a binding affinity for plasmin and thrombin [76]. Another study evidenced an association between worse HL prognosis and high platelet count [77]. The estimated incidence of thrombosis among systemic HL patients is approximately 3–13% according to the stage of disease, compared with 0.1% in the general population [74]. The incidence rate (IR) of thrombosis is lower in HL (IR 4.7%) than in low-grade Non-Hodgkin’s lymphoma (NHL), IR 6.3%, and in high-grade NHL, IR 8.3% [78]. Etiology of thromboembolism is thought to be multifactorial, in part due to the tumor cells’ capacity to release coagulant molecules, and in part due to a deficiency, acquired or herediraty, of antithrombin III, protein S or C, or factor V Leiden [79,80,81,82].

There is evidence that HRS cells express both the pro-angiogenic vascular endothelial growth factor (VEGF) and its receptors, and that VEGF expression can be induced by hypoxia [83,84]. Moreover, VEGF in macrophages and in the extracellular matrix might facilitate HL progression, contributing to the pathophysiology and the metastasis of this malignancy [85]. PD-1 and PD-L1 association with pro-angiogenic proteins, including hypoxia-inducible factors (HIFs) and VEGF, have been reported in several malignancies [86,87]. In cHL, PD-L1 and PD-L2 expression well correlated with VEGF expression and micro-vessel density [88,89]. EBV infection also well correlated with VEGF level and poorer survival [90]. 

**Figure 3:**
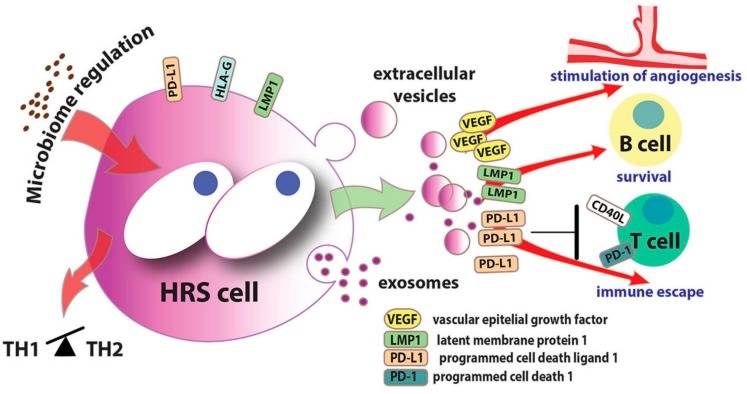
Interactions between the microbiome and Hodgkin Reed-Sternberg (HRS) cells and effects of the release of extracellular vesicles/exosomes into the Hodgkin lymphoma (HL microenvironment. Intestinal microbiota has been suggested to play a role in HL development by acting on the host immune system, for instance type 1 T helper cells (Th1). Exosomes and extracellular vesicles from HL and Epstein-Barr virus (EBV)-positive HL exacerbate the worst phenotype of the tumor by remodeling the tumor microenvironment trough the delivery of Vascular Epithelial Growth Factor (VEGF, which promotes micro-vessel density), Latent Membrane Protein 1 (LMP1, which activates various molecular pathways including B-cell survival), while Programmed Cell Death Ligand 1 (PD-L1) trough binding of the Programmed Cell Death 1 (PD-1) receptor induces immunosuppressive function of the T-cells.

A recent study reported that VEGF molecules can be released by extracellular vesicles in the tumor microenvironment and that EBVpositive HRS cells secrete exosomes to support tumor cell survival and HL progression [91] (Figure 3). It was demonstrated that exosomes, containing VEGF, EBV-miRNAs, and LMP1, are phagocytosed by monocytes in a process that regulates tumor supporting activities. Follicular dendritic cells also incorporated exosomes released from peri-follicular EBV-infected cells and therfore present LMP1 to EBV-negative germinal center preapoptotic centrocytes [92], the B-cells identified as the histogenesis origin of the HRS [93]. Inactivation of the proto-oncogene MYC, a key gene in HRS cells [94], was found to force PD-L1 expression in HRS, with the concomitant decreased in macrophages and CD8-positive T-cells number in the microenvironment, tumor progression, and maintenance of angiogenesis [13,95]. Thus, the co-expression of PD-L1 and VEGF is indicated as a potential prognostic factor in cHL. The combination of anti PD-1/PD-L1 and anti-VEGF drugs in addition to the conventional therapies should therefore be investigated. Accumulating evidences showed that exosomes from HL and EBV-positive HL exacerbate the worse phenotype of the tumor by remodeling the tumor microenvironment by carrying several important molecules (i.e., VEGF, LMP1, PD-L1), whose circulating concentration positively correlate with IFN-γ concentration and T-cell function (Figure 3). Accordantly to these results, the concentration of PD-L1 in circulating vesicles during IC inhibitor treatment could be considered a marker of the treatment response. On the other hand, the removal of these circulating vesicles could be a new minimally-invasive therapeutic approach [96].

## 7. Microbiome

The gut flora, also called microbiota, may modulate the cargo of antigen presentation to T-cells. In particular, microbiome (gene pool of microbiota) emerges from immune checkpoint studies from 2018. A study in this setting provided strong evidence that the efficacy of a PD-L1 inhibitor can be improved by the modulation of gut microbiota [97]. The efficacy of cancer immunotherapy diminished with the administration of antibiotics, while superior efficacy was observed in the presence of specific gut microbes such as *Bifidobacteria* spp., *Akkermansia muciniphila*, *Enterococcus hirae*, and *Bacteroides* spp., among others [98,99,100,101]. A few studies considered the gut microflora in HL. One of these found modest reductions in the diversity of fecal bacteria in young adult HL survivors compared with their unaffected twins [102]. Another study revealed that exposure to a low oral microbiome at early life increased the risk of developing HL ten-fold [103]. Germ-free mice showed a more pronounced pro-inflammatory Th2 cytokine profile compared to conventionally-raised animals [104]. Overall data indicate a potential role of gut microbiome in the development of HL, but unfortunately, so far, no data are available on HL patients treated with PD-1–PD-L1.

Despite the exciting findings in this research field, the underlying molecular mechanisms by which the gut bacterial species enhance PD-1 and PD-L1 blockade therapy remain largely unknown. Nonetheless, the use of bacteriophages has been proposed as a simple tool for eliminating unfavorable bacteria to enhance the efficacy of immunotherapy in these patients [105].

## 8. Duration of Therapy and Future Directions in the Use of Checkpoint Inhibitors

Although data from follow-up of trials using IC inhibitors are still limited in time, precluding the accurate estimation of OS and the assessment of the durability of response in long-term time. Disease progression of ~16 months in patients after pembrolizumab (anti–PD-1) treatment of refractory/relapsed cHL, though achieving an excellent response rate, indicated a non-durable long-term memory for anti-tumor immunologic response [31,106]. Consequently, patients need lifelong treatment until their disease progresses or unacceptable toxicity occur, however proposed for a time not exceeding 24 months [107]. Moreover, since, probably, some patients might gain benefit from a shorter treatment, research should investigate a strategy to select patients based on this aim. Another important question is if relapsed disease after discontinuing therapy may have a benefit of a PD-1 inhibitor retreatment. Thus, while IC inhibitors are currently approved in relapsed/progressive disease, further ongoing trials are necessary to evaluate the efficacy of IC inhibitors earlier in the course of the disease (e.g., as a pre-transplant salvage regimen or as part of the initial induction therapy). Thus, the best timing to initiate anti-PD-1 therapy and the best combination therapy remain, today, open questions [106].

To prolong the durability of the PD-1 response is proposed to block PD-L1 throughout the whole patient, not only to blockPD-1 on the tumor and T-cells. This is because the specialized microenvironment in cHL and the high abundance of secreted PD-L1 may obstruct the ability of immune cells to efficiently eliminate the tumor cells. Indeed, it is now recognized that PD-L1, which is known to be present on the cell surface not only of tumor cells but also of macrophages, may be also secreted into the bloodstream, so affecting cells in distant sites away from the tumor, not only cells in the tumor site. HRS cells have adapted multiple mechanisms to evade immune surveillance in an immune-rich milieu [106]. Another approach to moving beyond IC blockade is to use anti-PD-1 agents with brentuximab vedotin, an anti-CD30 targeted tumor cell therapy. When a tumor cell dies, it releases neo-antigens, which are then swallowed by macrophages and antigen-presenting cells and then activate T-cell response, thus immune function could be reactivated by IC inhibitors. Some studies using this approach have been completed and seem effective [107], but, also, in these cases the durability of response is not clearly definite, in part because most patients went on to transplant before. Based on the same rationale, the development of other components targeting both the tumor and/or immune cells is starting. For example, bispecific antibodies targeting both CD30 on HRS cells and CD16A on natural killer cells [108] or blocking CD47, suppressing macrophages phagocytosis [109] or even blocking CTLA-4, suppressing T-cell activation [110], and in combination with PD-1 blockade.

Combination IC inhibition against CTLA-4 and PD-1 or PD-L1 became a new option in various solid tumors. CTLA-4 (CD152), inhibits T-cell functions by indirectly diminishing signaling of the T-cell costimulatory receptor CD28 [111]. Both CD28 and CTLA-4 receptors bind CD80 and CD86, which are present on the surface of T-cells, but CTLA-4 does so with much higher affinity, effectively outcompeting CD28. Furthermore, CTLA-4 may also remove CD80 and CD86 from bystander cells by trans-endocytosis. Globally, the reduced CD28 availability increases the activation threshold of T-cells, thus dampening the recognition of tumor antigens. CTLA-4 is constitutively expressed by Tregs, but it can also be upregulated by other T-cell subsets, especially CD4-positive T-cells upon activation, as well as tumor cells. It is believed that CTLA-4 impacts on the stage of T-cell activation mainly in the draining lymph nodes by removing CD80/CD86 from the surface of antigen-presenting cells, thus decreasing the ability to effectively stimulate tumor-specific T cells. For T cell priming, dendritic cells load antigen from the tumor and transport it to the draining lymph nodes, where they present antigen/MHC complexes to T-cells, but T-cells become activated only if they are able to recognize both the complexes (through their T-cell receptors) and to bind CD80 and CD86 on DCs (through CD28 costimulatory receptors). When cells reach satisfactory levels of CD28/CD80/CD86 complexes, T-cells proliferate, increase their survival, produce growth cytokines such as interleukin-2, increase energy metabolism, and upregulate survival genes [112]. Activated T cells then migrate to the tumor site to kill malignant cells. Furthermore, accumulation of CTLA-4-positive Treg cells in the tumor microenvironment increases immunosuppressive function in the milieu around the tumor cells. In fact, CTLA-4 is located primarily in the intracellular compartment, but after T cell activation by antigen complex and CD28/CD80/CD86 binding, CTLA-4 is upregulated and transported to the cell surface where it is released into the microenvironment by exocytosis. Then the CTLA-4/CD80/CD86 link prevents further T cell activation. In contrast, PD-1 mainly affects the CD8-positive effectors T-cells and other immune cell responses, such as dendritic cells, B-cells, and perhaps other T-independent pathways to be elucidated [113,114]. Thus, the roles of CTLA-4 and PD-1 are largely distinct, with CTLA-4 regulating T cell proliferation early in an immune response and primarily in lymph nodes, while PD-1 represses T-cells later during the immune response and primarily in peripheral tissues. Thus CTLA-4 and PD-1 operate independently, although both act on T cell activated by TCR/antigen recognition, and as a result both are more effective in tumors that are infiltrated by T-cells and that have high mutation rates. Their independent modes of action provide the rationale for the combined administration of both anti CTLA-4 and anti-PD-1. Ipilimumab was the first CTLA-4 inhibitor to be tested and approved for the treatment of cancer patients [115]. Data on CTLA-4 inhibitors use in cHL have been reported for patients after autologous stem cell transplantation [116]. Combined inhibition of PD1/PD-L1- and CTLA4-mediated suppression in cHL was hypothesed by the finding that CTLA4 significantly increased during treatment with nivolumab and ipilimumab, and remained so after the end of treatment with this drug [117]. Although now tested only in melanoma, dual blockade of PD-1 (with nivolumab) and CTLA-4 (with ipilimumab) resulted in higher response rate and longer survival than in patients treated with a single IC inhibitor [118]. However, this combination strongly increased the adverse event rates in melanoma and small cell lung cancer, thus requiring special warnings and cautions for use [119,120]. The CheckMate-039 trial examined CTLA-4 and PD-1 combination in 31 patients with HL, and showed that efficacy and toxicity was similar to that of anti-PD-1 alone. A clinical trial of brentuximab vedotin and nivolumab, in combination with or without ipilimumab in patients with relapsed or refractory cHL is currently in progress (NCT01896999).

## Figures and Tables

**Table 1:** Approved PD-1/PD-L1 checkpoint inhibitors.

Antibody	Target	Approval Date
Nivolumab	PD-1	2014
Pembrolizumab	PD-1	2014
Atezolizumab	PD-L1	2016
Avelumab	PD-L1	2017
Durvalumab	PD-L1	2017
Cemiplimab	PD-1	2018
Tislelizumab	PD-1	2018
Sintilimab	PD-1	2018
Camrelizumab	PD-1	2018
Spartalizumab	PD-1	Expected in 2020

PD-1: programmed cell death 1; PD-L1: programmed cell death ligand 1.

## References

[1] Gloghini A., Bosco A., Ponzoni M., Spina M., Carbone A. (2015). Immunoarchitectural patterns in nodular lymphocyte predominant Hodgkin lymphoma: Pathologic and clinical implications. Expert Rev. Hematol..

[2] Swerdlow S.H., Campo E., Pileri S.A., Harris N.L., Stein H., Siebert R., Advani R., Ghielmini M., Salles G.A., Zelenetz A.D. (2016). The 2016 revision of the World Health Organization classification of lymphoid neoplasms. Blood.

[3] Küppers R., Engert A., Hansmann M.-L. (2012). Hodgkin lymphoma. J. Clin. Invest..

[4] Dolcetti R., Gloghini A., Caruso A., Carbone A. (2016). A lymphomagenic role for HIV beyond immune suppression?. Blood.

[5] De Re V., Boiocchi M., De Vita S., Dolcetti R., Gloghini A., Uccini S., Baroni C., Scarpa A., Cattoretti G., Carbone A. (1993). Subtypes of Epstein-Barr virus in HIV-1-associated and HIV-1-unrelated Hodgkin’s disease cases. Int. J. Cancer.

[6] Aldinucci D., Celegato M., Casagrande N. (2016). Microenvironmental interactions in classical Hodgkin lymphoma and their role in promoting tumor growth, immune escape and drug resistance. Cancer Lett..

[7] Jalali S., Price-Troska T., Bothun C., Villasboas J., Kim H.-J., Yang Z.-Z., Novak A.J., Dong H., Ansell S.M. (2019). Reverse signaling via PD-L1 supports malignant cell growth and survival in classical Hodgkin lymphoma. Blood Cancer J..

[8] Yan Y., Kumar A.B., Finnes H., Markovic S.N., Park S., Dronca R.S., Dong H. (2018). Combining Immune Checkpoint Inhibitors With Conventional Cancer Therapy. Front. Immunol..

[9] Simon S., Labarriere N. (2017). PD-1 expression on tumor-specific T cells: Friend or foe for immunotherapy?. Oncoimmunology.

[10] Butte M.J., Keir M.E., Phamduy T.B., Sharpe A.H., Freeman G.J. (2007). Programmed death-1 ligand 1 interacts specifically with the B7-1 costimulatory molecule to inhibit T cell responses. Immunity.

[11] Karwacz K., Bricogne C., MacDonald D., Arce F., Bennett C.L., Collins M., Escors D. (2011). PD-L1 co-stimulation contributes to ligand-induced T cell receptor down-modulation on CD8+ T cells. EMBO Mol. Med..

[12] Sugiura D., Maruhashi T., Okazaki I.-M., Shimizu K., Maeda T.K., Takemoto T., Okazaki T. (2019). Restriction of PD-1 function by cis-PD-L1/CD80 interactions is required for optimal T cell responses. Science.

[13] Garcia-Diaz A., Shin D.S., Moreno B.H., Saco J., Escuin-Ordinas H., Rodriguez G.A., Zaretsky J.M., Sun L., Hugo W., Wang X. (2017). Interferon Receptor Signaling Pathways Regulating PD-L1 and PD-L2 Expression. Cell Rep..

[14] Loke P., Allison J.P. (2003). PD-L1 and PD-L2 are differentially regulated by Th1 and Th2 cells. Proc. Natl. Acad. Sci. USA.

[15] Ghiotto M., Gauthier L., Serriari N., Pastor S., Truneh A., Nunès J., Olive D. (2010). PD-L1 and PD-L2 differ in their molecular mechanisms of interaction with PD-1. Int. Immunol..

[16] Latchman Y., Wood C.R., Chernova T., Chaudhary D., Borde M., Chernova I., Iwai Y., Long A.J., Brown J.A., Nunes R. (2001). PD-L2 is a second ligand for PD-1 and inhibits T cell activation. Nat. Immunol..

[17] Ansari M.J.I., Salama A.D., Chitnis T., Smith R.N., Yagita H., Akiba H., Yamazaki T., Azuma M., Iwai H., Khoury S.J. (2003). The Programmed Death-1 (PD-1) Pathway Regulates Autoimmune Diabetes in Nonobese Diabetic (NOD) Mice. J. Exp. Med..

[18] Zhang Y., Xu J., Hua J., Liu J., Liang C., Meng Q., Wei M., Zhang B., Yu X., Shi S. (2019). A PD-L2-based immune marker signature helps to predict survival in resected pancreatic ductal adenocarcinoma. J. Immunother. Cancer.

[19] Wang H., Yao H., Li C., Liang L., Zhang Y., Shi H., Zhou C., Chen Y., Fang J.-Y., Xu J. (2017). PD-L2 expression in colorectal cancer: Independent prognostic effect and targetability by deglycosylation. Oncoimmunology.

[20] Jung H.I., Jeong D., Ji S., Ahn T.S., Bae S.H., Chin S., Chung J.C., Kim H.C., Lee M.S., Baek M.-J. (2017). Overexpression of PD-L1 and PD-L2 Is Associated with Poor Prognosis in Patients with Hepatocellular Carcinoma. Cancer. Res. Treat..

[21] Gupta S., Vanderbilt C.M., Cotzia P., Arias-Stella J.A., Chang J.C., Zehir A., Benayed R., Nafa K., Razavi P., Hyman D.M. (2019). Next-Generation Sequencing-Based Assessment of JAK2, PD-L1, and PD-L2 Copy Number Alterations at 9p24.1 in Breast Cancer: Potential Implications for Clinical Management. J. Mol. Diagn..

[22] Baptista M.Z., Sarian L.O., Derchain S.F.M., Pinto G.A., Vassallo J. (2016). Prognostic significance of PD-L1 and PD-L2 in breast cancer. Hum. Pathol..

[23] Green M.R., Monti S., Rodig S.J., Juszczynski P., Currie T., O’Donnell E., Chapuy B., Takeyama K., Neuberg D., Golub T.R. (2010). Integrative analysis reveals selective 9p24.1 amplification, increased PD-1 ligand expression, and further induction via JAK2 in nodular sclerosing Hodgkin lymphoma and primary mediastinal large B-cell lymphoma. Blood.

[24] Green M.R., Rodig S., Juszczynski P., Ouyang J., Sinha P., O’Donnell E., Neuberg D., Shipp M.A. (2012). Constitutive AP-1 activity and EBV infection induce PD-L1 in Hodgkin lymphomas and posttransplant lymphoproliferative disorders: Implications for targeted therapy. Clin. Cancer Res..

[25] Ansell S.M. (2018). Hodgkin lymphoma: 2018 update on diagnosis, risk-stratification, and management. Am. J. Hematol..

[26] Ansell S.M. (2017). Nivolumab in the Treatment of Hodgkin Lymphoma. Clin. Cancer Res..

[27] Menzies A.M., Johnson D.B., Ramanujam S., Atkinson V.G., Wong A.N.M., Park J.J., McQuade J.L., Shoushtari A.N., Tsai K.K., Eroglu Z. (2017). Anti-PD-1 therapy in patients with advanced melanoma and preexisting autoimmune disorders or major toxicity with ipilimumab. Ann. Oncol..

[28] Xu-Monette Z.Y., Zhou J., Young K.H. (2018). PD-1 expression and clinical PD-1 blockade in B-cell lymphomas. Blood.

[29] Wu X., Gu Z., Chen Y., Chen B., Chen W., Weng L., Liu X. (2019). Application of PD-1 Blockade in Cancer Immunotherapy. Comput. Struct. Biotechnol. J..

[30] Younes A., Santoro A., Shipp M., Zinzani P.L., Timmerman J.M., Ansell S., Armand P., Fanale M., Ratanatharathorn V., Kuruvilla J. (2016). Nivolumab for classical Hodgkin’s lymphoma after failure of both autologous stem-cell transplantation and brentuximab vedotin: A multicentre, multicohort, single-arm phase 2 trial. Lancet Oncol..

[31] Chen R., Zinzani P.L., Fanale M.A., Armand P., Johnson N.A., Brice P., Radford J., Ribrag V., Molin D., Vassilakopoulos T.P. (2017). Phase II Study of the Efficacy and Safety of Pembrolizumab for Relapsed/Refractory Classic Hodgkin Lymphoma. J. Clin. Oncol..

[32] Sharma P., Hu-Lieskovan S., Wargo J.A., Ribas A. (2017). Primary, Adaptive, and Acquired Resistance to Cancer Immunotherapy. Cell.

[33] Ansell S.M. (2019). Immunotherapy in Hodgkin Lymphoma: The Road Ahead. Trends in Immunol..

[34] Radford J., Illidge T., Counsell N., Hancock B., Pettengell R., Johnson P., Wimperis J., Culligan D., Popova B., Smith P. (2015). Results of a trial of PET-directed therapy for early-stage Hodgkin’s lymphoma. N. Engl. J. Med..

[35] Shaverdian N., Lisberg A.E., Bornazyan K., Veruttipong D., Goldman J.W., Formenti S.C., Garon E.B., Lee P. (2017). Previous radiotherapy and the clinical activity and toxicity of pembrolizumab in the treatment of non-small-cell lung cancer: A secondary analysis of the KEYNOTE-001 phase 1 trial. Lancet Oncol..

[36] Quéro L., Gilardin L., Fumagalli I., Martin V., Guillerm S., Bauduceau O., Kirova Y.M., Hennequin C., Brice P. (2019). Anti-PD-1 immunotherapy in combination with sequential involved-site radiotherapy in heavily pretreated refractory Hodgkin lymphoma. Cancer Radiother..

[37] Nie J., Wang C., Liu Y., Yang Q., Mei Q., Dong L., Li X., Liu J., Ku W., Zhang Y. (2019). Addition of Low-Dose Decitabine to Anti-PD-1 Antibody Camrelizumab in Relapsed/Refractory Classical Hodgkin Lymphoma. J. Clin. Oncol..

[38] Chen Q., Li T., Yue W. (2018). Drug response to PD-1/PD-L1 blockade: Based on biomarkers. Onco Targets Ther..

[39] Curiel T.J., Wei S., Dong H., Alvarez X., Cheng P., Mottram P., Krzysiek R., Knutson K.L., Daniel B., Zimmermann M.C. (2003). Blockade of B7-H1 improves myeloid dendritic cell-mediated antitumor immunity. Nat. Med..

[40] He Y.-F., Zhang G.-M., Wang X.-H., Zhang H., Yuan Y., Li D., Feng Z.-H. (2004). Blocking programmed death-1 ligand-PD-1 interactions by local gene therapy results in enhancement of antitumor effect of secondary lymphoid tissue chemokine. J. Immunol..

[41] Peng D., Kryczek I., Nagarsheth N., Zhao L., Wei S., Wang W., Sun Y., Zhao E., Vatan L., Szeliga W. (2015). Epigenetic silencing of TH1-type chemokines shapes tumour immunity and immunotherapy. Nature.

[42] Jenkins R.W., Barbie D.A., Flaherty K.T. (2018). Mechanisms of resistance to immune checkpoint inhibitors. Br. J. Cancer..

[43] Arce-Gomez B., Jones E.A., Barnstable C.J., Solomon E., Bodmer W.F. (1978). The genetic control of HLA-A and B antigens in somatic cell hybrids: Requirement for beta2 microglobulin. Tissue Antigens.

[44] Peng W., Chen J.Q., Liu C., Malu S., Creasy C., Tetzlaff M.T., Xu C., McKenzie J.A., Zhang C., Liang X. (2016). Loss of PTEN Promotes Resistance to T Cell-Mediated Immunotherapy. Cancer Discov..

[45] Seliger B., Ferrone S. (2020). HLA Class I Antigen Processing Machinery Defects in Cancer Cells-Frequency, Functional Significance, and Clinical Relevance with Special Emphasis on Their Role in T Cell-Based Immunotherapy of Malignant Disease. Methods Mol. Biol..

[46] Liu Y.-W., Sun M., Xia R., Zhang E.-B., Liu X.-H., Zhang Z.-H., Xu T.-P., De W., Liu B.-R., Wang Z.-X. (2015). LincHOTAIR epigenetically silences miR34a by binding to PRC2 to promote the epithelial-to-mesenchymal transition in human gastric cancer. Cell Death Dis..

[47] Zappasodi R., Budhu S., Hellmann M.D., Postow M.A., Senbabaoglu Y., Manne S., Gasmi B., Liu C., Zhong H., Li Y. (2018). Non-conventional Inhibitory CD4+Foxp3-PD-1hi T Cells as a Biomarker of Immune Checkpoint Blockade Activity. Cancer Cell.

[48] Zou W., Wolchok J.D., Chen L. (2016). PD-L1 (B7-H1) and PD-1 pathway blockade for cancer therapy: Mechanisms, response biomarkers, and combinations. Sci. Transl. Med..

[49] Nijland M., Veenstra R.N., Visser L., Xu C., Kushekhar K., van Imhoff G.W., Kluin P.M., van den Berg A., Diepstra A. (2017). HLA dependent immune escape mechanisms in B-cell lymphomas: Implications for immune checkpoint inhibitor therapy?. Oncoimmunology.

[50] De Re V., Caggiari L., Mussolin L., D’Amore E.S., Famengo B., De Zorzi M., Martina L., Elia C., Pillon M., Santoro N. (2017). HLA-G+3027 polymorphism is associated with tumor relapse in pediatric Hodgkin’s lymphoma. Oncotarget.

[51] Caocci G., Greco M., Fanni D., Senes G., Littera R., Lai S., Risso P., Carcassi C., Faa G., La Nasa G. (2016). HLA-G expression and role in advanced-stage classical Hodgkin lymphoma. Eur. J. Histochem..

[52] Da Silva P.B., Real J.M., Ferreira L.R.P., Esteves G.H., Brito F. (2018). do N.; Baiocchi, O.C.G. Soluble PD-1 and PD-L1 as potential biomarkers for classical Hodgkin lymphoma. Hematol. Oncol..

[53] Castro F., Cardoso A.P., Gonçalves R.M., Serre K., Oliveira M.J. (2018). Interferon-Gamma at the Crossroads of Tumor Immune Surveillance or Evasion. Front. Immunol..

[54] Goodman A.M., Kato S., Bazhenova L., Patel S.P., Frampton G.M., Miller V., Stephens P.J., Daniels G.A., Kurzrock R. (2017). Tumor Mutational Burden as an Independent Predictor of Response to Immunotherapy in Diverse Cancers. Mol. Cancer Ther..

[55] Ahmadzadeh M., Johnson L.A., Heemskerk B., Wunderlich J.R., Dudley M.E., White D.E., Rosenberg S.A. (2009). Tumor antigen-specific CD8 T cells infiltrating the tumor express high levels of PD-1 and are functionally impaired. Blood.

[56] Postow M.A., Sidlow R., Hellmann M.D. (2018). Immune-Related Adverse Events Associated with Immune Checkpoint Blockade. N. Engl. J. Med..

[57] Saruwatari K., Sato R., Nakane S., Sakata S., Takamatsu K., Jodai T., Mito R., Horio Y., Saeki S., Tomita Y. (2019). The Risks and Benefits of Immune Checkpoint Blockade in Anti-AChR Antibody-Seropositive Non-Small Cell Lung Cancer Patients. Cancers.

[58] Horio Y., Takamatsu K., Tamanoi D., Sato R., Saruwatari K., Ikeda T., Nakane S., Nakajima M., Saeki S., Ichiyasu H. (2018). Trousseau’s syndrome triggered by an immune checkpoint blockade in a non-small cell lung cancer patient. Eur. J. Immunol..

[59] Tomita Y., Sueta D., Kakiuchi Y., Saeki S., Saruwatari K., Sakata S., Jodai T., Migiyama Y., Akaike K., Hirosako S. (2017). Acute coronary syndrome as a possible immune-related adverse event in a lung cancer patient achieving a complete response to anti-PD-1 immune checkpoint antibody. Ann. Oncol..

[60] Malissen N., Lacotte J., Du-Thanh A., Gaudy-Marqueste C., Guillot B., Grob J.-J. (2017). Macrophage activation syndrome: A new complication of checkpoint inhibitors. Eur. J. Cancer.

[61] Borghaei H., Paz-Ares L., Horn L., Spigel D.R., Steins M., Ready N.E., Chow L.Q., Vokes E.E., Felip E., Holgado E. (2015). Nivolumab versus Docetaxel in Advanced Nonsquamous Non-Small-Cell Lung Cancer. N. Engl. J. Med..

[62] Brahmer J., Reckamp K.L., Baas P., Crinò L., Eberhardt W.E.E., Poddubskaya E., Antonia S., Pluzanski A., Vokes E.E., Holgado E. (2015). Nivolumab versus Docetaxel in Advanced Squamous-Cell Non-Small-Cell Lung Cancer. N. Engl. J. Med..

[63] Herbst R.S., Baas P., Kim D.-W., Felip E., Pérez-Gracia J.L., Han J.-Y., Molina J., Kim J.-H., Arvis C.D., Ahn M.-J. (2016). Pembrolizumab versus docetaxel for previously treated, PD-L1-positive, advanced non-small-cell lung cancer (KEYNOTE-010): A randomised controlled trial. Lancet.

[64] Rittmeyer A., Barlesi F., Waterkamp D., Park K., Ciardiello F., von Pawel J., Gadgeel S.M., Hida T., Kowalski D.M., Dols M.C. (2017). Atezolizumab versus docetaxel in patients with previously treated non-small-cell lung cancer (OAK): A phase 3, open-label, multicentre randomised controlled trial. Lancet.

[65] Antonia S.J., Villegas A., Daniel D., Vicente D., Murakami S., Hui R., Yokoi T., Chiappori A., Lee K.H., de Wit M. (2017). Durvalumab after Chemoradiotherapy in Stage III Non-Small-Cell Lung Cancer. N. Engl. J. Med..

[66] Weber J.S., Kähler K.C., Hauschild A. (2012). Management of immune-related adverse events and kinetics of response with ipilimumab. J. Clin. Oncol..

[67] Wang P.-F., Chen Y., Song S.-Y., Wang T.-J., Ji W.-J., Li S.-W., Liu N., Yan C.-X. (2017). Immune-Related Adverse Events Associated with Anti-PD-1/PD-L1 Treatment for Malignancies: A Meta-Analysis. Front Pharmacol..

[68] Rudzki J.D. (2018). Management of adverse events related to checkpoint inhibition therapy. Memo.

[69] Champiat S., Dercle L., Ammari S., Massard C., Hollebecque A., Postel-Vinay S., Chaput N., Eggermont A., Marabelle A., Soria J.-C. (2017). Hyperprogressive Disease Is a New Pattern of Progression in Cancer Patients Treated by Anti-PD-1/PD-L1. Clin. Cancer Res..

[70] Frelaut M., Le Tourneau C., Borcoman E. (2019). Hyperprogression under Immunotherapy. Int. J. Mol. Sci..

[71] Falanga A., Donati M.B. (2001). Pathogenesis of thrombosis in patients with malignancy. Int. J. Hematol..

[72] Korte W. (2008). Cancer and thrombosis: An increasingly important association. Support. Care Cancer.

[73] Repetto O., Maiero S., Magris R., Miolo G., Cozzi M.R., Steffan A., Canzonieri V., Cannizzaro R., De Re V. (2018). Quantitative Proteomic Approach Targeted to Fibrinogen β Chain in Tissue Gastric Carcinoma. Int. J. Mol. Sci..

[74] Kwaan H.C., Vicuna B. (2007). Incidence and pathogenesis of thrombosis in hematologic malignancies. Semin. Thromb. Hemost..

[75] Borchmann S., Müller H., Haverkamp H., Baues C., Marková J., Hüttmann A., Glunz A., Fuchs M., Borchmann P., Engert A. (2019). Symptomatic osteonecrosis as a treatment complication in Hodgkin lymphoma: An analysis of the German Hodgkin Study Group (GHSG). Leukemia.

[76] Repetto O., Mussolin L., Elia C., Martina L., Bianchi M., Buffardi S., Sala A., Burnelli R., Mascarin M., De Re V. (2018). Proteomic Identification of Plasma Biomarkers in Children and Adolescents with Recurrent Hodgkin Lymphoma. J. Cancer.

[77] Farruggia P., Puccio G., Sala A., Todesco A., Buffardi S., Garaventa A., Bottigliero G., Bianchi M., Zecca M., Locatelli F. (2016). The prognostic value of biological markers in paediatric Hodgkin lymphoma. Eur. J. Cancer.

[78] Caruso V., Di Castelnuovo A., Meschengieser S., Lazzari M.A., de Gaetano G., Storti S., Iacoviello L., Donati M.B. (2010). Thrombotic complications in adult patients with lymphoma: A meta-analysis of 29 independent cohorts including 18 018 patients and 1149 events. Blood.

[79] Hathaway W.E., Hays T. (1975). Hypercoagulability in childhood cancer. J. Pediatr. Surg..

[80] Bick R.L. (1978). Alterations of hemostasis associated with malignancy: Etiology, pathophysiology, diagnosis and management. Semin. Thromb. Hemost..

[81] Dutcher J.P. (1987). Hematologic abnormalities in patients with nonhematologic malignancies. Hematol. Oncol. Clin. North Am..

[82] Shapiro A.D., Clarke S.L., Christian J.M., Odom L.F., Hathaway W.E. (1993). Thrombosis in children receiving L-asparaginase. Determining patients at risk. Am. J. Pediatr. Hematol. Oncol..

[83] Doussis-Anagnostopoulou I.A., Talks K.L., Turley H., Debnam P., Tan D.C., Mariatos G., Gorgoulis V., Kittas C., Gatter K.C. (2002). Vascular endothelial growth factor (VEGF) is expressed by neoplastic Hodgkin-Reed-Sternberg cells in Hodgkin’s disease. J. Pathol..

[84] Marinaccio C., Nico B., Maiorano E., Specchia G., Ribatti D. (2014). Insights in Hodgkin Lymphoma angiogenesis. Leuk. Res..

[85] Zerdes I., Matikas A., Bergh J., Rassidakis G.Z., Foukakis T. (2018). Genetic, transcriptional and post-translational regulation of the programmed death protein ligand 1 in cancer: Biology and clinical correlations. Oncogene.

[86] Ramjiawan R.R., Griffioen A.W., Duda D.G. (2017). Anti-angiogenesis for cancer revisited: Is there a role for combinations with immunotherapy?. Angiogenesis.

[87] Liu S., Qin T., Jia Y., Li K. (2019). PD-L1 Expression Is Associated With VEGFA and LADC Patients’ Survival. Front. Oncol..

[88] Koh Y.W., Han J.-H., Yoon D.H., Suh C., Huh J. (2017). PD-L1 expression correlates with VEGF and microvessel density in patients with uniformly treated classical Hodgkin lymphoma. Ann. Hematol..

[89] Linke F., Harenberg M., Nietert M.M., Zaunig S., von Bonin F., Arlt A., Szczepanowski M., Weich H.A., Lutz S., Dullin C. (2017). Microenvironmental interactions between endothelial and lymphoma cells: A role for the canonical WNT pathway in Hodgkin lymphoma. Leukemia.

[90] Koh Y.W., Han J.-H., Yoon D.H., Suh C., Huh J. (2018). Epstein-Barr virus positivity is associated with angiogenesis in, and poorer survival of, patients receiving standard treatment for classical Hodgkin’s lymphoma. Hematol. Oncol..

[91] Higuchi H., Yamakawa N., Imadome K.-I., Yahata T., Kotaki R., Ogata J., Kakizaki M., Fujita K., Lu J., Yokoyama K. (2018). Role of exosomes as a proinflammatory mediator in the development of EBV-associated lymphoma. Blood.

[92] Uccini S., Al-Jadiry M.F., Cippitelli C., Talerico C., Scarpino S., Al-Darraji A.F., Al-Badri S.A.F., Alsaadawi A.R., Al-Hadad S.A., Ruco L. (2018). Burkitt lymphoma in Iraqi children: A distinctive form of sporadic disease with high incidence of EBV+ cases and more frequent expression of MUM1/IRF4 protein in cases with head and neck presentation. Pediatr. Blood Cancer.

[93] Küppers R., Klein U., Schwering I., Distler V., Bräuninger A., Cattoretti G., Tu Y., Stolovitzky G.A., Califano A., Hansmann M.-L. (2003). Identification of Hodgkin and Reed-Sternberg cell-specific genes by gene expression profiling. J. Clin. Invest..

[94] Tiacci E., Döring C., Brune V., van Noesel C.J.M., Klapper W., Mechtersheimer G., Falini B., Küppers R., Hansmann M.-L. (2012). Analyzing primary Hodgkin and Reed-Sternberg cells to capture the molecular and cellular pathogenesis of classical Hodgkin lymphoma. Blood.

[95] Casey S.C., Tong L., Li Y., Do R., Walz S., Fitzgerald K.N., Gouw A.M., Baylot V., Gütgemann I., Eilers M. (2016). MYC regulates the antitumor immune response through CD47 and PD-L1. Science.

[96] Chen G., Huang A.C., Zhang W., Zhang G., Wu M., Xu W., Yu Z., Yang J., Wang B., Sun H. (2018). Exosomal PD-L1 contributes to immunosuppression and is associated with anti-PD-1 response. Nature.

[97] Routy B., Le Chatelier E., Derosa L., Duong C.P.M., Alou M.T., Daillère R., Fluckiger A., Messaoudene M., Rauber C., Roberti M.P. (2018). Gut microbiome influences efficacy of PD-1-based immunotherapy against epithelial tumors. Science.

[98] Viaud S., Saccheri F., Mignot G., Yamazaki T., Daillère R., Hannani D., Enot D.P., Pfirschke C., Engblom C., Pittet M.J. (2013). The intestinal microbiota modulates the anticancer immune effects of cyclophosphamide. Science.

[99] Ida N., Dzutsev A., Stewart C.A., Smith L., Bouladoux N., Weingarten R.A., Molina D.A., Salcedo R., Back T., Cramer S. (2013). Commensal bacteria control cancer response to therapy by modulating the tumor microenvironment. Science.

[100] Daillère R., Vétizou M., Waldschmitt N., Yamazaki T., Isnard C., Poirier-Colame V., Duong C.P.M., Flament C., Lepage P., Roberti M.P. (2016). Enterococcus hirae and Barnesiella intestinihominis Facilitate Cyclophosphamide-Induced Therapeutic Immunomodulatory Effects. Immunity.

[101] Zitvogel L., Ma Y., Raoult D., Kroemer G., Gajewski T.F. (2018). The microbiome in cancer immunotherapy: Diagnostic tools and therapeutic strategies. Science.

[102] Cozen W., Yu G., Gail M.H., Ridaura V.K., Nathwani B.N., Hwang A.E., Hamilton A.S., Mack T.M., Gordon J.I., Goedert J.J. (2013). Fecal microbiota diversity in survivors of adolescent/young adult Hodgkin lymphoma: A study of twins. Br. J. Cancer.

[103] Cozen W., Hamilton A.S., Zhao P., Salam M.T., Deapen D.M., Nathwani B.N., Weiss L.M., Mack T.M. (2009). A protective role for early oral exposures in the etiology of young adult Hodgkin lymphoma. Blood.

[104] Olszak T., An D., Zeissig S., Vera M.P., Richter J., Franke A., Glickman J.N., Siebert R., Baron R.M., Kasper D.L. (2012). Microbial exposure during early life has persistent effects on natural killer T cell function. Science.

[105] Międzybrodzki R., Borysowski J., Weber-Dąbrowska B., Fortuna W., Letkiewicz S., Szufnarowski K., Pawełczyk Z., Rogóż P., Kłak M., Wojtasik E. (2012). Clinical aspects of phage therapy. Adv. Virus Res..

[106] Meti N., Esfahani K., Johnson N.A. (2018). The Role of Immune Checkpoint Inhibitors in Classical Hodgkin Lymphoma. Cancers.

[107] Vassilakopoulos T.P., Chatzidimitriou C., Asimakopoulos J.V., Arapaki M., Tzoras E., Angelopoulou M.K., Konstantopoulos K. (2019). Immunotherapy in Hodgkin Lymphoma: Present Status and Future Strategies. Cancers.

[108] Rothe A., Sasse S., Topp M.S., Eichenauer D.A., Hummel H., Reiners K.S., Dietlein M., Kuhnert G., Kessler J., Buerkle C. (2015). A phase 1 study of the bispecific anti-CD30/CD16A antibody construct AFM13 in patients with relapsed or refractory Hodgkin lymphoma. Blood.

[109] Ansell S., Chen R.W., Flinn I.W., Maris M.B., O’Connor O.A., Johnson L.D., Irwin M., Petrova P.S., Uger R.A., Sievers E.L. (2016). A Phase 1 Study of TTI-621, a Novel Immune Checkpoint Inhibitor Targeting CD47, in Patients with Relapsed or Refractory Hematologic Malignancies. Blood.

[110] Rotte A. (2019). Combination of CTLA-4 and PD-1 blockers for treatment of cancer. J. Exp. Clin. Cancer Res..

[111] Vardhana S., Younes A. (2016). The immune microenvironment in Hodgkin lymphoma: T cells, B cells, and immune checkpoints. Haematologica.

[112] Buchbinder E.I., Desai A. (2016). CTLA-4 and PD-1 Pathways. Am. J. Clin. Oncol..

[113] Thibult M.-L., Mamessier E., Gertner-Dardenne J., Pastor S., Just-Landi S., Xerri L., Chetaille B., Olive D. (2013). PD-1 is a novel regulator of human B-cell activation. Int. Immunol..

[114] Xiao X., Lao X.-M., Chen M.-M., Liu R.-X., Wei Y., Ouyang F.-Z., Chen D.-P., Zhao X.-Y., Zhao Q., Li X.-F. (2016). PD-1hi Identifies a Novel Regulatory B-cell Population in Human Hepatoma That Promotes Disease Progression. Cancer Discov..

[115] Phan G.Q., Yang J.C., Sherry R.M., Hwu P., Topalian S.L., Schwartzentruber D.J., Restifo N.P., Haworth L.R., Seipp C.A., Freezer L.J. (2003). Cancer regression and autoimmunity induced by cytotoxic T lymphocyte-associated antigen 4 blockade in patients with metastatic melanoma. Proc. Natl. Acad. Sci. USA.

[116] Bashey A., Medina B., Corringham S., Pasek M., Carrier E., Vrooman L., Lowy I., Solomon S.R., Morris L.E., Holland H.K. (2009). CTLA4 blockade with ipilimumab to treat relapse of malignancy after allogeneic hematopoietic cell transplantation. Blood.

[117] Greil R., Pleyer L., Jansko B., Feierabend C., Rettenbacher L., Stiefel O., Rass C., Morre P., Neureiter D., Greil-Ressler S. (2018). Sequential immunotherapy in a patient with primary refractory Hodgkin lymphoma and novel mutations. Oncotarget.

[118] Valsecchi M.E. (2015). Combined Nivolumab and Ipilimumab or Monotherapy in Untreated Melanoma. N. Engl. J. Med..

[119] Hodi F.S., Chiarion-Sileni V., Gonzalez R., Grob J.-J., Rutkowski P., Cowey C.L., Lao C.D., Schadendorf D., Wagstaff J., Dummer R. (2018). Nivolumab plus ipilimumab or nivolumab alone versus ipilimumab alone in advanced melanoma (CheckMate 067): 4-year outcomes of a multicentre, randomised, phase 3 trial. Lancet Oncol..

[120] Antonia S.J., López-Martin J.A., Bendell J., Ott P.A., Taylor M., Eder J.P., Jäger D., Pietanza M.C., Le D.T., de Braud F. (2016). Nivolumab alone and nivolumab plus ipilimumab in recurrent small-cell lung cancer (CheckMate 032): A multicentre, open-label, phase 1/2 trial. Lancet Oncol..

